# Patient engagement with surgical site infection prevention: an expert panel perspective

**DOI:** 10.1186/s13756-017-0202-3

**Published:** 2017-05-12

**Authors:** E. Tartari, V. Weterings, P. Gastmeier, J. Rodríguez Baño, A. Widmer, J. Kluytmans, A. Voss

**Affiliations:** 1 Infection Control Program and WHO Collaborating Centre on Patient Safety, Geneva University Hospitals and Faculty of Medicine, Geneva, Switzerland; 2 Laboratory for Microbiology and Infection Control, Amphia Hospital, Breda, The Netherlands; 3 Department of Medical Microbiology, Radboud University Medical Centre, Nijmegen, The Netherlands; 4 Institute of Hygiene and Environmental Medicine, Charite ´ University Medicine in Berlin, Berlin, Germany; 5 Unidad de Gestión Clínica de Enfermedades Infecciosas y Microbiología, Hospital Universitario Virgen Macarena/Instituto de Biomedicina de Sevilla (IBiS)/CSIC/Universidad de Sevilla, Seville, Spain; 6 University Hospital and University of Basel, Division of infectious diseases & hospital epidemiology, Basel, Switzerland; 7 Julius Center for Health Sciences and Primary Care, UMC Utrecht, Utrecht, The Netherlands; 8 Medical Microbiology and Infectious Diseases, Canisius-Wilhelmina Hospital, Nijmegen, The Netherlands

**Keywords:** Surgical site infection, Surgical wound infection, Patient participation, Empowered patient, Patient involvement, Patient education, Surgery, Hair removal, *Staphylococcus aureus*, MRSA, Screening, Decolonization, Hand hygiene, Smoking, Diabetes mellitus, Wound care, Surgical site care bundle, Infection control

## Abstract

Despite remarkable developments in the use of surgical techniques, ergonomic advancements in the operating room, and implementation of bundles, surgical site infections (SSIs) remain a substantial burden, associated with increased morbidity, mortality and healthcare costs. National and international recommendations to prevent SSIs have been published, including recent guidelines by the World Health Organization, but implementation into clinical practice remains an unresolved issue. SSI improvement programs require an integrative approach with measures taken during the pre-, intra- and postoperative care from the numerous stakeholders involved. The current SSI prevention strategies have focused mainly on the role of healthcare workers (HCWs) and procedure related risk factors. The importance and influence of patient participation is becoming an increasingly important concept and advocated as a means to improve patient safety. Novel interventions supporting an active participative role within SSI prevention programs have not been assessed. Empowering patients with information they require to engage in the process of SSI prevention could play a major role for the implementation of recommendations. Based on available scientific evidence, a panel of experts evaluated options for patient involvement in order to provide pragmatic recommendations for pre-, intra- and postoperative activities for the prevention of SSIs. Recommendations were based on existing guidelines and expert opinion. As a result, 9 recommendations for the surgical patient are presented here, including a practice brief in the form of a patient information leaflet. HCWs can use this information to educate patients and allow patient engagement.

**Electronic supplementary material:**

The online version of this article (doi:10.1186/s13756-017-0202-3) contains supplementary material, which is available to authorized users.

## Background

Surgical site infections (SSIs) continue to constitute a major challenge to healthcare institutions as a leading cause of healthcare associated infections (HAIs) [1]. The detrimental consequences of SSIs are associated with poorer patient outcomes, affecting mortality, morbidity and increasing healthcare associated expenditure [2, 3]. SSIs are the most common cause of HAIs in income-poor settings and the second most common cause of HAIs in resource-rich countries [4, 5]. Data from low and middle-income countries showed an overall incidence rate of 5.6%, whilst 2.6% reported in the USA and 1.6% in Germany [5–7]. Yet, a large part of HAIs, including SSIs is preventable.

There are various factors contributing to the risk of SSI occurrence and preventative measures require an integrative approach that focuses through the pre-, intra- and postoperative care involving all the stakeholders. Numerous multimodal preventive intervention programs based on guidelines, surgical site care bundles, and surgical safety checklists have been established [8–10]. Despite several advancements in procedures, the optimal reduction of SSIs remains a challenge. To date, most SSI preventive measures have focused on the surgical team with patient participation intervention on SSI prevention unexplored hence, effectiveness of this intervention remains to be assessed [11]. In the past few years, active engagement of the patient in the processes of healthcare has gained momentum on a global scale as a means to improve patient safety [12]. There has been a greater emphasis and a marked emergence of more active patients, who are characterized as being better informed, having a more participatory role and being involved in decision-making process concerning their health [13, 14]. Patient participation in SSI preventive programs refers to interventions ranging from patient education to ensure a clean skin pre- surgery to empowering patients to remind HCWs to not shave the surgical site; to perform hand hygiene prior contact with the surgical site; to assist in preserving a warm body temperature throughout surgery and other actions outlined here. This strategy might be useful but its effectiveness has not been researched.

We aimed to identify basic and pragmatic recommendations for patients to be empowered by healthcare workers (HCWs) to seek information at an early stage and actively engage throughout their surgical journey. We analyzed several international guidelines that are considered standards at a global level and identified various effective elements of pre-, intra- and postoperative care where patient can play an important role to ensure that preventative measures have been implemented. The primary audience for these recommendations is the surgical patient and HCWs including infection control specialists, surgeons, nurses and others providing direct patient care, as the basis for developing patient education material and supporting patients throughout their surgical journey with SSI preventive measures.

### Methods for the development of recommendations

Five key infection prevention and control experts and infectious disease specialists, with a special interest in surgical site infections formed the expert panel (PG, JRB, AW, JK, AV), and convened a day-long meeting to discuss the topic. All members are scientifically renown in peer-reviewed journals and have made essential contributions in the development of international guidelines for the prevention of surgical site infections.

In the absence of evidence on patient participation in SSI prevention, a systematic literature review was not possible. International published guidelines on pre-, intra- and postoperative measures for SSI prevention, mainly from the World Health Organization (WHO), Centers for Disease Control and Prevention (CDC) and their reference lists were searched to identify relevant studies [10, 15, 16]. Of note, for the recently published WHO SSI prevention guidelines, 27 systematic reviews were conducted to support the evidence of published recommendations [15, 16].

To this end, the panel identified 9 fundamental recommendations based on evidence and on expert advice and consensus when evidence was scarce. Based on the recommendations clear and pragmatic implementation strategies were developed in the form of a patient’s information leaflet containing proactive information patients require to engage in the prevention of their surgical site infection during the pre-, intra- and postoperative care (Additional file 1). This information is intended to enhance the knowledge of the surgical patient towards SSI prevention thus inviting the patient to actively engage in the processes of care. The practical recommendations can be adapted by healthcare facilities in different patient education formats. The leaflet was pretested for readability among individuals of patient representative groups and adjusted according to the remarks provided. The recommendations and the implementation strategy summarized in brief action points for patients to participate during their surgical journey are presented in Table 1.

**Table 1:** Summary of recommendations and key actions for patients to participate in SSI prevention program

Recommendations	Key actions for patients
1. *Staphylococcus aureus* screening and decolonization	For high risk surgery, nasal screening for methicillin-sensitive *S. aureus* (MSSA) or methicillin-resistant *S. aureus* (MRSA) carriage is recommended
	Decolonization treatment with mupirocin 2% ointment with or without a combination of chlorhexidine gluconate body wash prior to surgery is needed for nasal carriage of MSSA or MRSA
	Apply decolonization treatment at least the night and the morning before your surgery Decolonization treatment for 3–5 days before the surgery if possible
2. Smoking	Inform your doctor about your smoking history before surgery
	Quit smoking 4 weeks or longer before your surgery
3. Hair removal	Shaving is strongly discouraged
	Do not remove hair at the site of the planned incision when at home Hair should only be removed with an electrical clipper
4. Hand hygiene	Clean your hands before eating a meal; after visiting the toilet or using commode/urinal; before and after touching your drip (IV line) or drainage bag/tube
	Visitors should not touch your wound or dressings
	Speak up if you do not see HCWs clean their hands
5. Body temperature	Ask about the procedures followed to keep you warm throughout surgery
	Take a hot shower shortly before the surgery
	Avoid “cooling down”
	Ask for extra blankets to keep yourself warm during transportation
	Speak up, if you feel cold before or after surgery and ask for a blanket
6. Preoperative showering and bathing	Make sure your skin is clean before surgery
	Shower or bathe (full body) with either soap (antimicrobial or non-antimicrobial) on the night before and/or in the morning of the surgery
7. Diabetes mellitus	See your doctor at least one month before your scheduled surgery
	Maintain stable blood glucose levels before, during and after surgery
	Inform HCWs about your routine insulin regime
8. Wound care after surgery	The wound dressing should be kept in place for 48 h after surgery
	If change of dressing is necessary, this should be done under a clean technique
	Ensure that HCWs clean hands immediately before changing your dressing
	Visitors should not touch your wound or the dressing when visiting you
	Make sure you know and understand how to care for your wound before leaving the hospital
	Report any redness, pain, swelling or fever to HCWs
9. Multidrug-resistant organism risk (MDRO)	Inform HCWs about any travel history or previous recent hospitalisation
	Inform of any known carriage of any MDRO such as MRSA, Extended Spectrum *β*-Lactamase (ESBL) producing or Carbapenem-resistant Enterobacteriaceae (CRE)

### Education opportunities to improve patient engagement

The information content provided here in a leaflet format has the potential to be translated in other languages and adaptable to local circumstances for use in multiple forms of communication and patient education strategies (Additional file 1). Educational interventions are likely to be more effective if they are multifaceted and broadly applicable to meet various health literacy needs across the general population [17]. The evolving healthcare system is marked by the emergence of more active patients and the patient is recognized as a key stakeholder, requiring more information that facilitates a participatory role [18]. Surgeons, nurses and other HCWs might consider various educational programs (written material, illustrations, images, computer technology, smartphone mobile applications, serious games, audio, video and demonstrations) to meet different learning needs and achieve active patient engagement. Utilization of social media and contemporary internet based resources has grown rapidly in the last decade with platforms such as Facebook, Twitter, YouTube, and blogs as emerging resources for patients seeking health information [19]. However, lack of quality information drives individuals to seek information from untrusted sources [14]. Such platforms may offer novel ways to share information on prevention of SSIs well in advance prior to the surgery before the patient is admitted to the hospital during pre- hospital admission visits, telephone conversations and repeated at different intervals throughout a patient’s surgical care pathway (i.e on admission, immediately before surgery, after surgery and prior to discharge from a facility). This information can be made easily accessible on the facility websites, on electronic devices given to patients and other formats, whilst providing trusted resources and empowering patients with information regarding their care.

### Healthcare workers’ role to encourage patient engagement

HCWs behavior can have a major effect and their support is crucial for successful patient engagement activities surrounding SSI prevention. Importantly, HCWs need to acknowledge that an active participatory role throughout the surgical journey may have an influence on patient action to prevent SSI. From in-depth focus groups with patients, Rawson et al. described that HCWs are failing to engage their patients with the decision-making process surrounding infections and their management thus, leading to misinformation, frustration and anxiety [14]. Poor communications by HCWs, low health literacy, lack of knowledge and quality of information of the subject have been reported as main obstacles to patient engagement driving individuals to seek information from untrusted online sources [14, 20, 21]. The detailed patient information provided here is designed in an easy to understand language surrounding patient activities to prevent the occurrence of SSI following surgery. Nonetheless, when providing education to patients on the recommendations presented here, HCWs must be mindful of patients’ health literacy needs, ensuring that this proactive information is adopted to meet the individual needs and that it is understandable. Emphasis is placed on encouraging an educational environment that stimulates patients to participate in their surgical care, by getting involved in a discussion, inviting and allowing time for questions and clarifications on the information provided. A recent systematic literature review has confirmed the importance of HCWs encouragement as a key component empowering patients to take action on the recommended safety behaviors [22].

### Recommendation 1: *Staphylococcus aureus* screening and decolonization

#### What can the patient do?

- If you are undergoing high risk surgery including cardiothoracic and orthopedic surgery ask your healthcare worker for a nasal screening test to identify methicillin-sensitive *Staphylococcus aureus* (MSSA) or methicillin-resistant *Staphylococcus aureus* (MRSA) carriage
- If you are a nasal carrier of MSSA or MRSA you should receive decolonization treatment with intranasal applications of mupirocin 2% ointment with or without a combination of chlorhexidine gluconate (CHG) antiseptic body wash prior to surgery
- The treatment should be applied at least the night and the morning before your surgery. If possible you should receive decolonization treatment for 3–5 days before the surgery. In this case, the decolonization treatment can be done at home and you should be provided with information or a patient information leaflet on the correct use of the treatment application method.

#### Rationale

*Staphylococcus aureus* nasal carriage increases a patient’s risk for developing a healthcare-associated infection with this microorganism, at least after cardiothoracic and orthopedic surgery [3, 23, 24]. Preoperative screening for nasal carriage and subsequent treatment of carriers with mupirocin ointment and chlorhexidine gluconate washes, reduces the risk for the development of hospital-acquired *S. aureus* infections by 79% for deep infections and 55% for superficial infections [25]. Also, the mean duration of hospital stay is reduced in treated carriers by approximately 2 days. A cost benefit analysis shows that the strategy is cost-effective and saves lives [25].

Mupirocin nasal ointment is an effective, safe and relatively cheap treatment for the eradication of carriage. Nevertheless mupirocin resistance has been reported, and therefore mupirocin should be used wisely for the recommended period of time [23, 25]. Alternatives to mupirocin and chlorhexidine gluconate (CHG) are currently under investigation.

Some facilities have experienced problems with the implementation of screening and instead perform universal decolonization preoperatively in the absence of screening [9, 26]. As the majority of patients do not carry *S. aureus* and treatment in this group is not effective, this is not the optimal strategy and should be considered with caution as it may contribute to mupirocin resistance. However, it is considered a better alternative than no decolonization at all.

### Recommendation 2: Smoking

#### What can the patient do?

- Inform your doctor about your smoking history well in advance prior to your surgery
- Quit smoking 4 weeks or longer before your surgery. Ask for nicotine replacement to help you stop smoking at least temporarily

#### Rationale

Smoking is a recognised independent risk factor for surgical site infections [27]. Smokers have a higher incidence of complications after surgery compared with non-smokers across all surgical specialties. Former smokers appear to have a lifetime higher risk of healing complications compared with patients who never smoked. Current smokers have increased rates of respiratory complications such as postoperative pneumonia and SSIs. Smoking increases other complications such as wound hematoma, discharge, or dehiscence in the immediate postoperative period. Smoking affects the normal wound healing process and may increase the risk of developing SSIs [27, 28]. Factors responsible for the increased risk of postoperative complications include: nicotine, nitric oxide, and carbon monoxide. Smoking causes endothelial dysfunction, inflammation, and progression of atherothrombotic disease. Consequently, smoking cessation is recommended in the preoperative period. This seems to be an effective measure to reduce postoperative complications even if it is introduced as late as four weeks before surgery [27].

### Recommendation 3: Hair removal

#### What can the patient do?

- At home, do not remove hair at the site of the planned incision (even if asked to do so). The skin may experience microscopic cuts and abrasions that microorganisms can enter and colonize these cuts. If you are shaving on a regular basis you will need to stop shaving near the surgical area at least five days before your surgery to prevent superficial infections
- While being in the hospital, ask the healthcare worker in charge of you, if any hair removal is deemed necessary. If you have local anesthesia or if someone wants to remove hair at the planned incision site using a razor, speak up. Shaving is strongly discouraged. If necessary, hair should only be removed with an electrical clipper [29]

#### Rationale

The hair removal procedure has been performed historically, because it is thought that the presence of hair can interfere with the exposure of the incision, the suturing of the incision and the application of adhesive wound dressings [30]. Nonetheless, hair does not need to be removed preoperatively unless the hair in or around the incision site interferes with the operation. While the data for the timing of hair removal are less convincing, in general, hair removal should be done as shortly before the operation, as possible. The preferred method of hair removal is by using a clipper [29, 30]. Using a razor can irritate the skin and lead to micro lesions. Consequently, microorganisms can progressively colonize the affected skin and thus significantly increase (double) the chance of a postoperative infection. While depilating creams are probably comparable to clippers in regard to postoperative wound infections, some patients may experience skin irritation and in general the procedure seems less practical and requires additional cleaning with water afterwards [29, 30].

### Recommendation 4: Hand hygiene

#### What can the patient do?

- Clean your hands by using an alcohol-based hand rub or, if your hands are visibly dirty, soap and water.
  - before eating a meal
  - after visiting the bathroom or using commode/urinal
  - before and after touching your wound or wound dressing
  - before and after touching your drip (IV line) or drainage bag/tube
- Make sure that healthcare workers clean their hands before assessing your wound, preferably with an alcohol-based hand rub solution
- Speak up if you do not see healthcare worker clean their hands before touching you
- Visitors should not touch your wound or dressings. If they need to be involved in wound care they should follow the same preventive measures as healthcare workers

#### Rationale

As much as 50–70% of all healthcare-associated infections are transmitted through the hands of HCWs due to lack of adherence to good hand hygiene practice [31, 32]. Appropriate hand hygiene of HCWs remains the most effective strategy to protect patients from healthcare-associated infections and limit the spread of antimicrobial resistant bacteria [31, 33, 34]. Yet, compliance with hand hygiene among HCWs is persistently substandard with an average of only 38.7% (range 5–89%) [12, 33].

There is strong evidence supporting implementing hand hygiene activities using multimodal strategies to improve compliance and reduce healthcare associated infections [33–36]. Additionally, involving patients to remind HCWs about their hand hygiene could lead to a sustained increase in compliance when combined with other multimodal strategies. McGuckin et al. showed that when patients were educated on admission to remind staff to clean their hands, hand hygiene practices of HCWs improved significantly, with a marked increase in soap consumption from 34 to 94% [37]. Other studies have shown a beneficial effect from improved hand hygiene practices in patients [38–40]. In a before-and-after retrospective study, Gagne et al. implemented a program to promote hand hygiene of patients and visitors, showing a 51% reduction in healthcare associated MRSA infections [38].

### Recommendation 5: Body temperature

#### What can the patient do?

- It is important that you do not cool down before and during the surgical procedure
- Ask your doctor or nurse about the procedures followed to keep you warm throughout surgery
- Take a hot shower shortly before the surgery is scheduled and stay under the cover after your shower, so as to preserve warm body temperature
- Avoid “cooling down” and do not put on the surgical gown and stay uncovered long before the surgery commences
- Ask for extra blankets to keep yourself warm during transportation from the ward to and from the operating room
- Speak up, if you feel cold before or after surgery and ask for a blanket

#### Rationale

Mild perioperative hypothermia, which is common during surgery, may increase patients’ susceptibility to perioperative surgical site infections by causing vasoconstriction and impaired immunity [41].

Despite an ongoing discussion within the scientific community, there is evidence of reduced rates of SSIs when a stable body temperature is maintained (core temperatures near 36.0–36.5 °C) and this so called “normothermia”, is part of many guidelines to prevent surgical site infections [42]. In short duration, for a clean surgical procedure, this can be achieved by active warming 30 min prior to surgery; in abdominal surgery, intra-operative active warming (e.g. forced-air warming blanket) has shown to reduce the rate of surgical site infections [43].

Less is known about “passively keeping warm”. While “passively keeping warm” was not scientifically compared to “unintentional cooling down”, experts believe that all measures should be taken to ensure that a warm body temperature is maintained. A hot shower shortly before the operation may relieve tension or even ease anxiety (due to oxytocin release) but the patient’s overall body temperature might drop if the patient is not covered immediately afterwards. In general, patients should avoid “cooling down” e.g. by changing into hospital gowns and laying on top of their bed long in advance before surgery. Patients should ask for an extra blanket when feeling cold and should maintain a warm body temperature even during transportation and at the holding bay of the operation theatre.

### Recommendation 6: Preoperative showering and bathing

#### What can the patient do?

- Make sure your skin is clean before you are due for surgery
- Shower or bathe (full body) with either soap (antimicrobial or non-antimicrobial) or an antiseptic agent on the night before and/or in the morning of the day you are scheduled for surgery

#### Rationale

The rationale behind whole body bathing or showering before surgery is to make the skin as clean as possible by removing the transient flora and some resident flora. Showering with an antiseptic reduces the amount of bacteria found on the skin but the effect on surgical site infections in the scientific evidence remains inconclusive [44]. There is no specific recommendation favoring one antiseptic agent over another. Some product may cause hypersensitivity or skin irritation for specific patients therefore an alternative product might be needed. There are no benefits for using body wipes or disposable disinfectant washcloths as compared to shower with un-medicated bar soap have been reported in the evidence for the prevention of surgical site infections. Also there is no specific recommendation in terms of optimal timing for showering prior to surgery, the total amount of soap or antiseptic application [44, 45].

### Recommendation 7: Diabetes mellitus

#### What can the patient do?

- If you have diabetes see your doctor at least one month before your scheduled surgery
- It is crucial to maintain stable blood glucose levels before, during and after surgery
- When hospitalised inform your doctor or nurse about your routine insulin regime

#### Rationale

Hyperglycemia is significantly associated with an increased risk for SSIs. Blood glucose levels rise during and after surgery due to surgical stress. Hyperglycemia impairs numerous host defense mechanisms and the risk of SSI increases [46, 47]. It is essential to control serum blood glucose levels for all surgical patients, including patients without diabetes. Well-controlled blood glucose levels have a significant benefit in reducing the risk of SSI development.

### Recommendation 8: Wound care after surgery

#### What can the patient do?

- The wound dressing should be kept in place for 48 h after surgery unless indications such as bleeding/exudate or abnormal pain are present
- If there is excess wound leakage and a change of dressing is necessary, this should be done under a clean technique (aseptic technique)
- Ensure that healthcare worker performs hand hygiene (clean hands) and puts gloves on immediately before changing your dressing
- Visitors should not touch your wound or the dressing when visiting you. If they need to help, they need to follow the mentioned infection prevention measures.
- Make sure you know and understand how to care for your wound before leaving the hospital
- If any symptoms of wound infection are present (redness, pain, swelling, fever) inform your doctor or nurse

#### Rationale

The wound is covered with a sterile dressing following surgery and while the wound is healing. Based on best practice and expert opinion, the wound should remain covered for 48 h following surgery, as this is period where initial healing over the wound takes place [48]. A surgical wound dressing is important to absorb leakage and to protect from microorganisms. There is no recommendation towards a particular dressing type; however a dressing such as semi permeable film, which is in use, would be appropriate [49]. It is important to change the surgical wound dressing under specific actions that prevent transmission of microorganisms. These require preparation of a surface area that prevents touch contamination of equipment; HCWs use gloves and apron and do not touch any other equipment in the surrounding environment.

### Recommendation 9: Multidrug-resistant organism (MDRO) risk

#### What can the patient do?

Inform your doctor:

- About your travel history within the last year or previous recent hospitalisation abroad, in particular if you have been recently hospitalised in countries within Southern and Eastern Europe, Middle East and North Africa (since these countries are recognized as high risk for MDRO)
- Known carriage of any MDRO such as MRSA, Extended Spectrum *β*-Lactamase (ESBL) producing or Carbapenem-resistant Enterobacteriaceae (CRE)

#### Rationale

Healthcare-associated infections are a major patient safety issue worldwide. The pace of increase in life expectancies and the ageing population is accompanied by greater prevalence of chronic diseases among hospitalised patients [50]. This, together with an increased use of diagnostics and therapeutic procedures affecting the host defenses will pose a significant challenge for the prevention of healthcare associated infections in the future [51]. Furthermore, resolving the threat presented by antimicrobial resistance (AMR) remains a challenge for healthcare systems threatening patient safety and leading to increased use of broad-spectrum antibiotics [52]. Controlling multidrug-resistant organisms (MDROs) is important because MDROs are resistant to usual antimicrobial therapy, increase patient morbidity and mortality, add to the cost of treatment, have the potential to spread and act as a reservoir of resistance genes for the transmission to other organisms. While MDRO is a global phenomenon, there are significant regional differences in terms of prevalence and patients who have been hospitalised in healthcare institutions abroad, are more likely to be colonized with MDRO [52, 53].

Patients with infections or carriers of pathogenic/resistant microorganisms admitted to hospital are potential sources of infection for patients and HCWs. Thus emphasizing the importance of surveillance and adherence to infection prevention and control policies for these patients [52, 53]. Of note, admission screening of patients at high risk of MDRO carriage (e.g. patients who have been previously hospitalised in healthcare institutions abroad) allows for additional transmission based precautions (e.g. contact precautions, single rooms) and efforts to limit the spread of MDROs.

## Conclusions

The impact of SSIs both on patients and healthcare organizations is profound; therefore efforts should focus on implementing diverse multidisciplinary prevention strategies. Patient engagement in preventing SSI might be an effective and useful strategy adding to the already existent surgical site care bundles. Yet, this topic is still at its infancy and deserves further rigorous studies to support the effectiveness of patient focused interventions in preventing surgical site infections. The elements recommended here, require further testing to define the optimal “bundle” that is effective and is regarded as acceptable part of quality improvement by HCWs and patients as well. Furthermore, patient engagement has the potential to help implementing current SSI guidelines into routine clinical practice. This aspect should be addressed through improving interventions that support patient education and encourage an active participatory role throughout the surgical care.

## Additional file

Additional file 1: A Patient Information Leaflet. (PDF 319 kb)

## Abbreviations

AMR: Antimicrobial resistance
CDC: Centers for disease control and prevention
CRE: Carbapenem-resistant Enterobacteriaceae
ESBL: Extended spectrum ß-Lactamase
HAI: Healthcare associated infection
HCWs: Healthcare workers
MDRO: Multidrug-resistant organisms
MRSA: Methicillin-resistance *Staphylococcus aureus*
MSSA: Methicillin-sensitive *Staphylococcus aureus*
SSI: Surgical site infections
WHO: World Health Organization
