# Supplementary material for: Patient engagement with surgical site infection prevention: an expert panel perspective
Source: Antimicrob Resist Infect Control. 2017 May 12;6:45. doi: 10.1186/s13756-017-0202-3 (PMC5427557; doi:10.1186/s13756-017-0202-3)
Supplement: Additional file 1: — A Patient Information Leaflet. (PDF 319 kb) [file 13756_2017_202_MOESM1_ESM.pdf]

PREVENTING SURGICAL WOUND INFECTIONS

A PATIENT INFORMATION LEAFLET

What is a Surgical Wound Infection?

Surgical wound infections are wound infections that occur after invasive surgical procedure at the body part where surgery has been performed. These infections may involve only the skin, or may be more serious and involve tissue under the skin or organs.

Wound infections may cause symptoms such as: redness, warmth, pain or tenderness around the affected site, discharge of pus or fever. The majority of wound infections become apparent within 30 days from the operation.

Surgical wound infection can often be prevented if care is taken before, during and after surgery.

What are hospitals doing to prevent the occurrence of surgical wound infections?

Hospitals collect information for specific operations and they can compare the findings to national infection rates.

- **Ask your doctor or nurse if the hospital participates in a surgical wound infection registration programme?**

For heart surgery, knee and hip surgery or other high risk surgery you may be tested for *Staphylococcus aureus.* This germ can cause serious skin or wound infections. The test involves rubbing a cotton-tipped swab in your nost rils.

- **If this germ is present, you will need to apply an ointment in your nost rils and possibly an antiseptic wash for the recommended duration before and after the operation.**

You may be prescribed antibiotics to further reduce

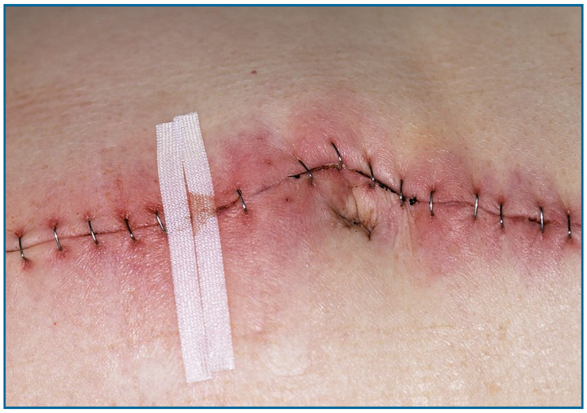

the risk of developing an infection. In most cases, antibiotics will be administered within 60 minutes before the surgery starts and should not last for longer than 24 hours following surgery.

What can I do to prevent surgical wound infections?

<u>Before the surgery:</u>

Smoking is a known risk factor linked with complications during and also after the operation. People who smoke are prone to developing more infections after surgery.

- **If your surgery is within the next 4 weeks stop smoking immediately. Otherwise stop smoking at least 4 weeks before the operation. Ask for medical support to help you stop smoking at least temporarily.**

Your doctor or nurse should be informed of the following:

- **Your medical history, particularly in case of diabetes mellitus.**
- **Your travel history within the last year or recent hospitalisation abroad.**
- **A known history of resistant bacteria.**

Shaving can cause skin cuts and abrasions, which may lead to a surgical wound infection.

- **At home, do not remove hair at the site of the planned incision (even if asked to do so). If you are shaving on a regular basis you need to stop shaving near the surgical area at least five days before your surgery.**
- **In the hospital, if hair needs to be removed, this should be done on the day of the operation using electric hair clippers with a disposable head.**

On the day of the surgery

- Have a **hot shower or bath** using plain soap or an antiseptic soap, on the night before your surgery and/or in the morning on the day you are scheduled for the surgery.
- After the shower or bath, **stay warm under the bed covers** so as to preserve the correct body temperature.
- Ask for **additional blankets** that will keep you warm during transportation from the ward to and from the operating room.
- Wear the gown given to you as close to the surgery as possible, to **avoid getting cold**.

After surgery

- Take special care to mobilise and get out of bed as early as possible after your surgery. **Early mobilisation prevents postoperative complications**.
- Following surgery you will find thin tubes (catheters) placed in blood vessels, or tubes inserted into the body such as a urinary catheter, necessary for the post-operative management.
  Check daily with your doctor if he presence of these catheters is still required. This will reduce the risk of developing a catheter related infection
- The wound dressing should **not be removed during the first 48 hours** after your surgery unless indicated otherwise (soiled or the edges of the wound dressing are open).
- During the first 48 hours after surgery, the wound should **stay dry.**
- Your doctor or nurse should take special care when changing your wound dressing. They should **clean their hands** either by using alcohol based hand rub or soap and water before any contact with you or before assessing your wound .
- Speak up if you do not see your doctor or nurse clean their hands before touching you.
- Ask your visitors **not to visit you if they are feeling unwell** (coughing, sneezing, fever, diarrhoea, vomiting).

Before being discharged/ at home:

- Ask your doctor or nurse for the necessary **contact details** in case you start feeling sick after you are discharged.
- Obtain all the necessary **information** on how to take care of your wound when at home .
- Always **clean your hands** before and after touching your wound or changing the wound dressing.
- If family members help with wound care they should clean their hands before touching your wound.
- Bath, sauna or swimming is not recommended during the first two weeks following surgery.

Report immediately to your doctor or nurse any symptoms of wound infection such as: redness, pain, drainage at the surgery site or fever.
